# Sciatic denervation differentially remodels skeletal muscle and deep fascia in the lower limb of male mice

**DOI:** 10.14814/phy2.71063

**Published:** 2026-08-20

**Authors:** Shun Otsuka, Yuki Tamaki, Kanae Umemoto, Naoyuki Hatayama, Munekazu Naito

**Affiliations:** ^1^ Department of Anatomy Aichi Medical University School of Medicine Nagakute Aichi Japan

**Keywords:** deep fascia, denervation, extracellular matrix, immobilization

## Abstract

The deep fascia surrounds skeletal muscles and contributes to force transmission and mechanical coordination, but its response to denervation remains unclear. This study investigated morphological and transcriptional adaptations of the deep fascia following sciatic nerve denervation in mice. Male C57BL/6J mice (*n* = 5–6/group) underwent unilateral sciatic nerve denervation or sham surgery. Fourteen days later, tibialis anterior (TA) muscle and overlying deep fascia (anterior crural fascia) were analyzed by histology and quantitative PCR. Denervation induced significant TA muscle atrophy with upregulation of inflammatory (*Il6*, *p* = 0.001), profibrotic (*Tgfb*, *p* < 0.001), and extracellular matrix (ECM)–related genes (*Mmp2*, *p* = 0.006; *Col3a1*, *p* = 0.005), and downregulation of regenerative factors (*Fgf2*, *p* < 0.001). Deep fascia thickness increased approximately 1.5‐fold (*p* = 0.012) and was accompanied by elevated *Il6* (*p* < 0.001), *Tgfb* (*p* = 0.035), *Col1a1* (*p* = 0.006), and *Col3a1* (*p* = 0.003) expression and a higher proportion of collagen type III (*p* = 0.015). In conclusion, sciatic nerve denervation induces muscle atrophy and deep fascia thickening accompanied by extracellular matrix remodeling. These findings demonstrate coordinated yet tissue‐specific remodeling of skeletal muscle and deep fascia following sciatic nerve denervation.

## Graphical abstract

Sciatic denervation induces differential remodeling of skeletal muscle and deep fascia, characterized by muscle atrophy and deep fascia thickening with distinct inflammatory and extracellular matrix responses.

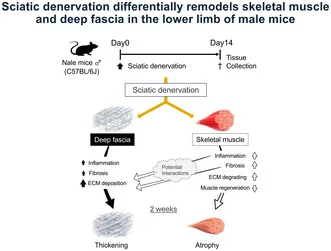

## 1 INTRODUCTION

The deep fascia is a dense connective tissue layer that envelopes skeletal muscles throughout the body (Benjamin, 2009; Kumka & Bonar, 2012; Stecco et al., 2008). It is anatomically continuous with skeletal muscles via the epimysium, forming a mechanical linkage that enables the transmission of contractile forces across adjacent muscles (Maas & Sandercock, 2010; Stecco et al., 2008; Yucesoy et al., 2008). Consistent with this functional coupling, structural properties of the deep fascia, including thickness, fiber orientation, and stiffness, are closely associated with muscle size and function (Otsuka et al., 2018, 2021; Stecco et al., 2014).

Skeletal muscle atrophy induced by denervation or physical inactivity is characterized by structural and molecular alterations, including inflammation and fibrosis, which impair force production and transmission (Madaro et al., 2018; Shen et al., 2019; Zhang et al., 2022). In addition to muscle, inactivity‐induced unloading affects surrounding connective tissues. For example, joint capsules undergo fibrotic changes following immobilization, leading to increased stiffness and reduced range of motion (Kaneguchi et al., 2020; Rebolledo et al., 2019; Sasabe et al., 2017). Similarly, disuse promotes extracellular matrix accumulation and connective tissue remodeling within skeletal muscle and its associated compartments (Csapo et al., 2020; Mavropalias et al., 2023; Sinha et al., 2020). These findings indicate that physical inactivity due to disuse or denervation induces coordinated structural changes in both muscle and adjacent connective tissues, which may contribute to impaired mobility and functional decline.

Given its close anatomical and mechanical association with skeletal muscle, the deep fascia may also undergo adaptive or pathological changes in response to denervation. However, the deep fascia is increasingly recognized as a distinct connective tissue with unique cellular composition and rich innervation (Fede et al., 2022; Suarez‐Rodriguez et al., 2022), suggesting that it may exhibit tissue‐specific biological responses. It therefore remains unclear whether the deep fascia exhibits gene expression changes that parallel those of innervated skeletal muscle or instead displays tissue‐specific molecular adaptations. Clarifying this issue is essential for understanding the role of fascial tissue in movement impairment and recovery following periods of denervation.

We hypothesized that sciatic denervation alters the mechanical and biochemical environment of both skeletal muscle and deep fascia. Therefore, the aim of this study was to determine the morphological and molecular adaptations of the deep fascia following sciatic denervation in mice.

## 2 MATERIALS AND METHODS

### 2.1 Animals

Eight‐week‐old male C57BL/6J mice obtained from Japan SLC (Shizuoka, Japan) were used in this study. Mice were housed in a room with a constant temperature of 23°C–25°C and humidity of 50%–60%, with free access to standard food (ORIENTAL YEAST CO., LTD., Certificated Diet MF, Tokyo, Japan) and water. The mice were acclimatized for at least 1 week prior to the experiments. Animals were monitored daily for signs of pain or distress, and humane endpoints were implemented when necessary.

### 2.2 Mouse models of sciatic nerve denervation

**FIGURE 1:**
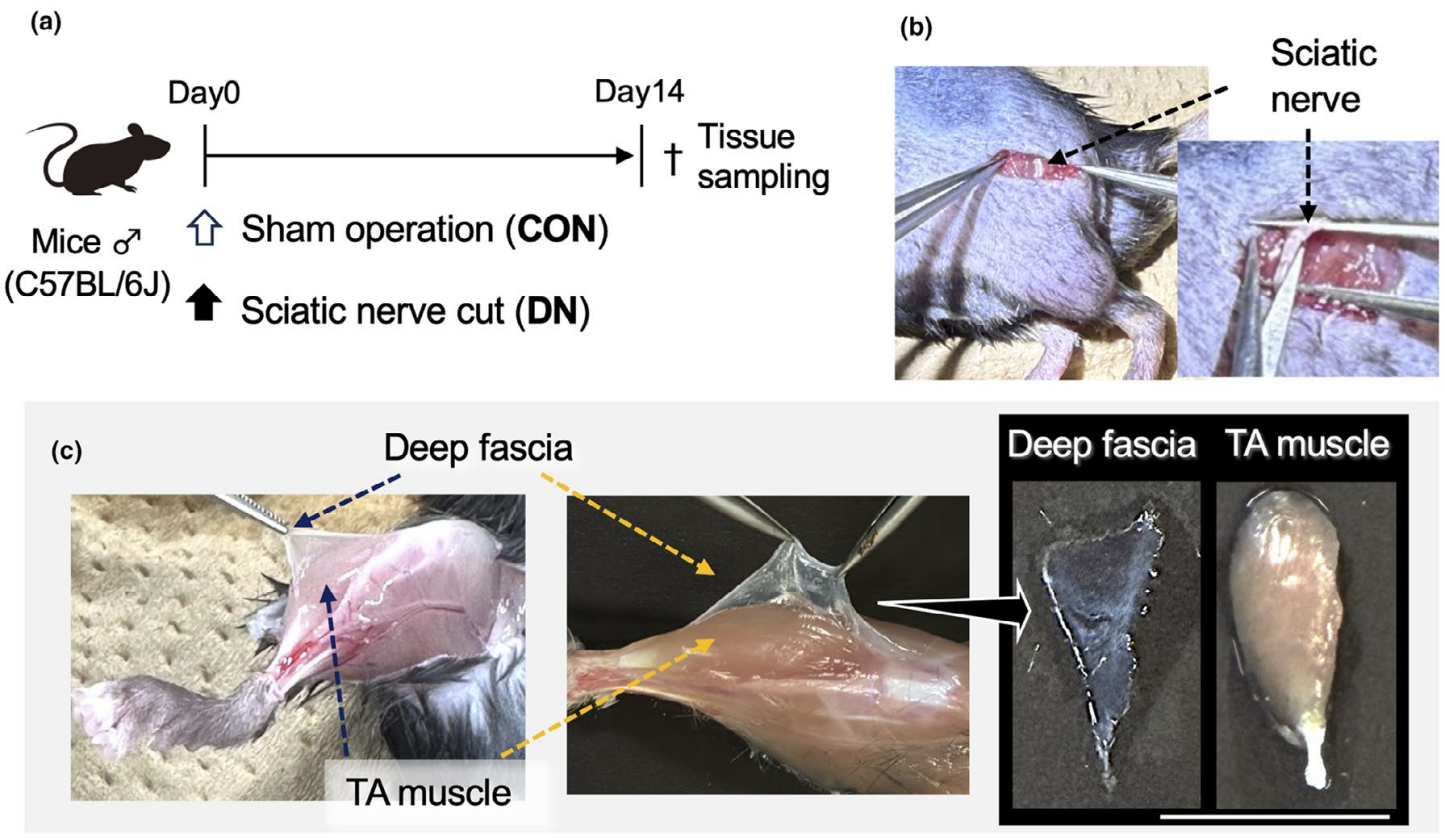
Experimental design and tissue collection. (a) Study timeline. Male C57BL/6J mice underwent sham operation (CON) or sciatic nerve transection (DN), and tissues were collected 14 days later. (b) Representative images of sciatic nerve transection. A segment of the left sciatic nerve was excised. (c) Representative image of the tibialis anterior (TA) muscle and overlying deep fascia (anterior crural fascia) used for gene expression analysis. Scale bar = 50 μm.

Mice were randomly assigned to one of two experimental groups: control (CON) and lower hindlimb restriction by sciatic nerve denervation (DN) groups (Figure 1a). For the CON group, mice underwent sham surgery in which the left sciatic nerve was bluntly exposed under isoflurane anesthesia (induction, 5%; maintenance, 2%), returned to its original position without transection, and the incision was closed. Postoperative analgesics were not administered to avoid potential effects on inflammatory and gene expression outcomes. In the DN group, mice were anesthetized with isoflurane, and the left sciatic nerve was bluntly exposed in a supine position while minimizing muscle damage. A 1‐mm segment of the nerve was excised to induce denervation of the lower limb muscles, as previously described (Menjo et al., 2024; Umezu et al., 2021) (Figure 1b). Fourteen days after the intervention, hindlimb tissues were harvested for weighing and gene expression analysis (*n* = 6/group), or histological staining (*n* = 5/group), as this time point corresponds to a phase of pronounced muscle atrophy and active transcriptional changes (Madaro et al., 2018; Umezu et al., 2021). We selected the tibialis anterior (TA) as the target muscle because it is directly covered by the deep fascia. The deep fascia analyzed in this study corresponded to the anterior crural fascia directly overlying the tibialis anterior muscle.

### 2.3 Histological analysis

At 14 days postintervention, mice were deeply anesthetized with isoflurane and euthanized by exsanguination during tissue collection. The left hindlimb was excised, fixed in 10% neutral‐buffered formalin (Japan Tanner Corporation, Cat. No. NBF000012, Osaka, Japan), decalcified with Kalkitox (FUJIFILM Wako Pure Chemical Corporation, Cat. No. 112–00651, Osaka, Japan), and neutralized in 5% sodium sulfate solution (FUJIFILM Wako Pure Chemical Corporation, Cat. No. 196–11,985) (*n* = 5/group). The tissues were dehydrated through a graded ethanol series and embedded in paraffin. Cross sections (4 μm thick) were cut at 30% of the proximal tibial length using a microtome and stained with Masson's trichrome (MT) and Picrosirius Red (PSR). Deep fascia thickness overlying the TA muscle was measured at 15 points (3 sections × 5 points) per sample under ×200 magnification using ImageJ software (NIH, Bethesda, MD, USA). Each point was measured twice, and the mean of the two measurements was calculated. The average of the 15 mean values was used as the representative thickness for each sample. All measurements were performed by a single investigator (S.O.). To assess intra‐observer reliability, the representative thickness values obtained from the first and second measurements were compared using the intraclass correlation coefficient (ICC; two‐way mixed‐effects model, absolute agreement, single measurements). The deep fascia was identified as the dense collagenous layer overlying the tibialis anterior muscle. Thickness was measured between the superficial and deep borders of the fascia while excluding subcutaneous connective tissue and epimysium. PSR‐stained sections were examined under polarized light microscopy using a BX53 microscope equipped with a DP74 digital camera, a UPlanXApo objective lens, and a U‐POT polarizing system (Olympus, Tokyo, Japan), and one section from each animal was analyzed. Images were converted to HSB color space using ImageJ (NIH), and hue histogram analysis was used to classify deep fascia into red (0–15), yellow (16‐39), and green (40–100) regions. Red–yellow signals were considered to represent thicker, more organized collagen fibers (predominantly type I collagen), whereas green signals were considered to indicate thinner and less organized fibers (associated with type III collagen) (Junqueira et al., 1978). Area fractions were quantified using color threshold‐based pixel analysis (Zerbinati & Calligaro, 2018).

### 2.4 Reverse transcription‐quantitative polymerase chain reaction (RT‐qPCR)

Following dissection, the wet weight of the left TA muscle was measured, and tissue was rapidly frozen for RNA extraction (*n* = 6/group). The contralateral TA muscle was also weighed to confirm the absence of detectable changes in the nondenervated limb but was not used as an experimental control. Total RNA was extracted separately from the isolated TA muscle and overlying deep fascia using TRIzol^®^ (Thermo Fisher Scientific, Cat. No. 15596018, Waltham, MA, USA) (Figure 1c). First‐strand cDNA synthesis was performed with the High‐Capacity cDNA reverse transcription kit (Thermo Fisher Scientific, Cat. No. A4368813). Quantitative polymerase chain reaction was performed using QuantStudio^®^ 3 (Thermo Fisher Scientific) using the TB Green Premix Ex Taq II (Tli RNaseH Plus, Cat. No. RR820D, Takara Bio, Shiga, Japan) master mix reagent and the ROX reference dye (Takara Bio, Cat. No. RR820D). Reaction conditions were as follows: 95°C for 30 s, followed by 40 cycles of 95°C for 5 s and 60°C for 31 s. Relative mRNA expression levels were calculated using the 2‐ΔΔCt method and normalized to *Gapdh*. Table S1 lists the primer sequences used for RT‐qPCR analysis of genes associated with inflammatory responses, profibrotic signaling, extracellular matrix deposition, and extracellular matrix remodeling in the present study.

### 2.5 Statistical analysis

All statistical analyses were performed using GraphPad Prism version 9.0 (GraphPad Software, San Diego, CA, USA). Data are presented as mean ± standard deviation (SD). Group comparisons were performed using an unpaired two‐tailed *t*‐test. A *p*‐value <0.05 was considered statistically significant. No correction for multiple comparisons was applied because each gene was treated as an independent, predefined biological endpoint.

## 3 RESULTS

### 3.1 TA muscle mass and deep fascia thickness after 14 days of sciatic denervation

**FIGURE 2:**
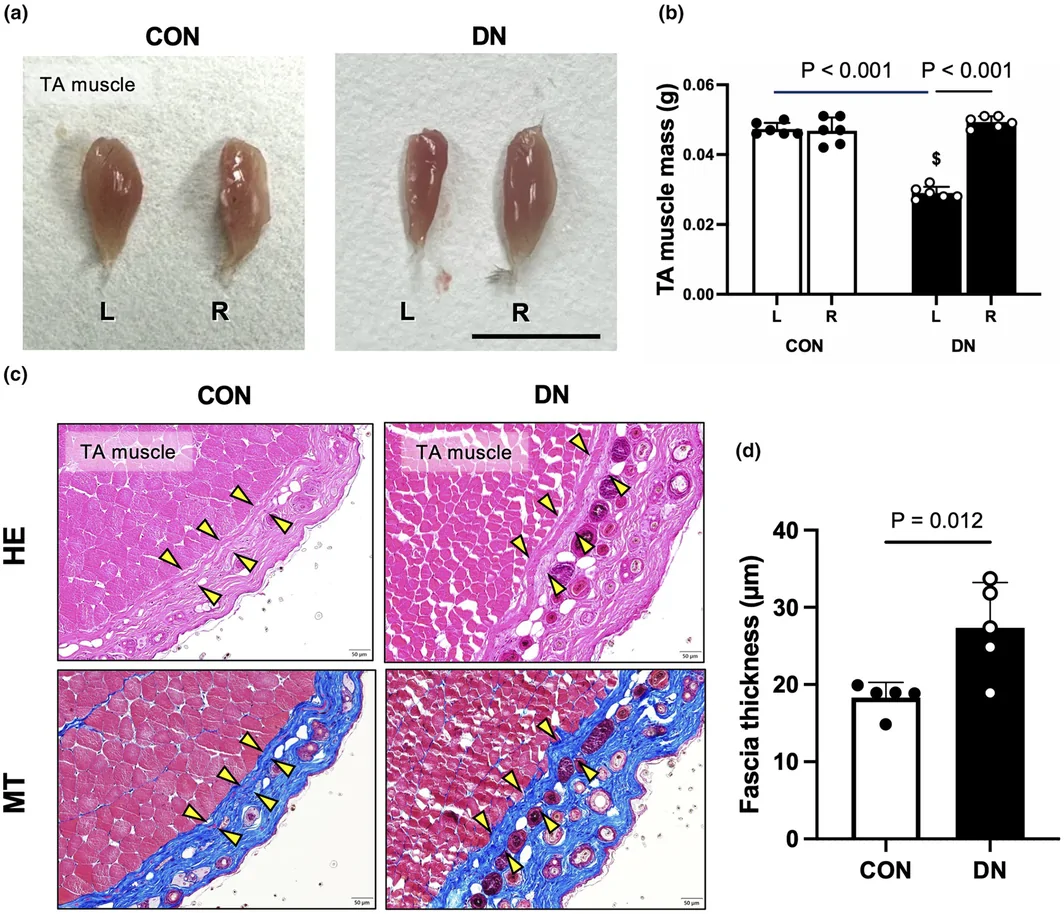
Effects of denervation on TA muscle mass and deep fascia thickness. (a) Representative images of tibialis anterior (TA) muscles from control (CON) and denervated (DN) mice. L, left (denervated) side; R, right (contralateral) side. Scale bar = 10 mm. (b) TA muscle mass in CON and DN groups. (c) Representative hematoxylin and eosin (HE) and Masson's trichrome (MT) staining of TA muscle and overlying deep fascia. Arrowheads indicate the deep fascia. Scale bar = 50 μm. (d) Quantification of deep fascia thickness. Data are presented as mean ± SD (*n* = 5–6/group).

The TA muscle mass on the left (denervated) side was significantly lower in the DN group than in the CON group (*p* < 0.001). No significant difference was observed in the right (contralateral side) TA muscle mass between the CON and DN groups (Figure 2a,b). MT staining revealed that the deep fascia on the left (denervated) side in the DN group (27.3 ± 5.9 μm) was thicker than in the CON group (18.3 ± 2.0 μm; *p* = 0.012) (Figure 2c,d). Intra‐observer reliability of fascia thickness measurements was excellent (ICC = 0.996) (Landis & Koch, 1977).

### 3.2 Gene expression changes in the deep fascia and skeletal muscle after 14 days of sciatic denervation

**FIGURE 3:**
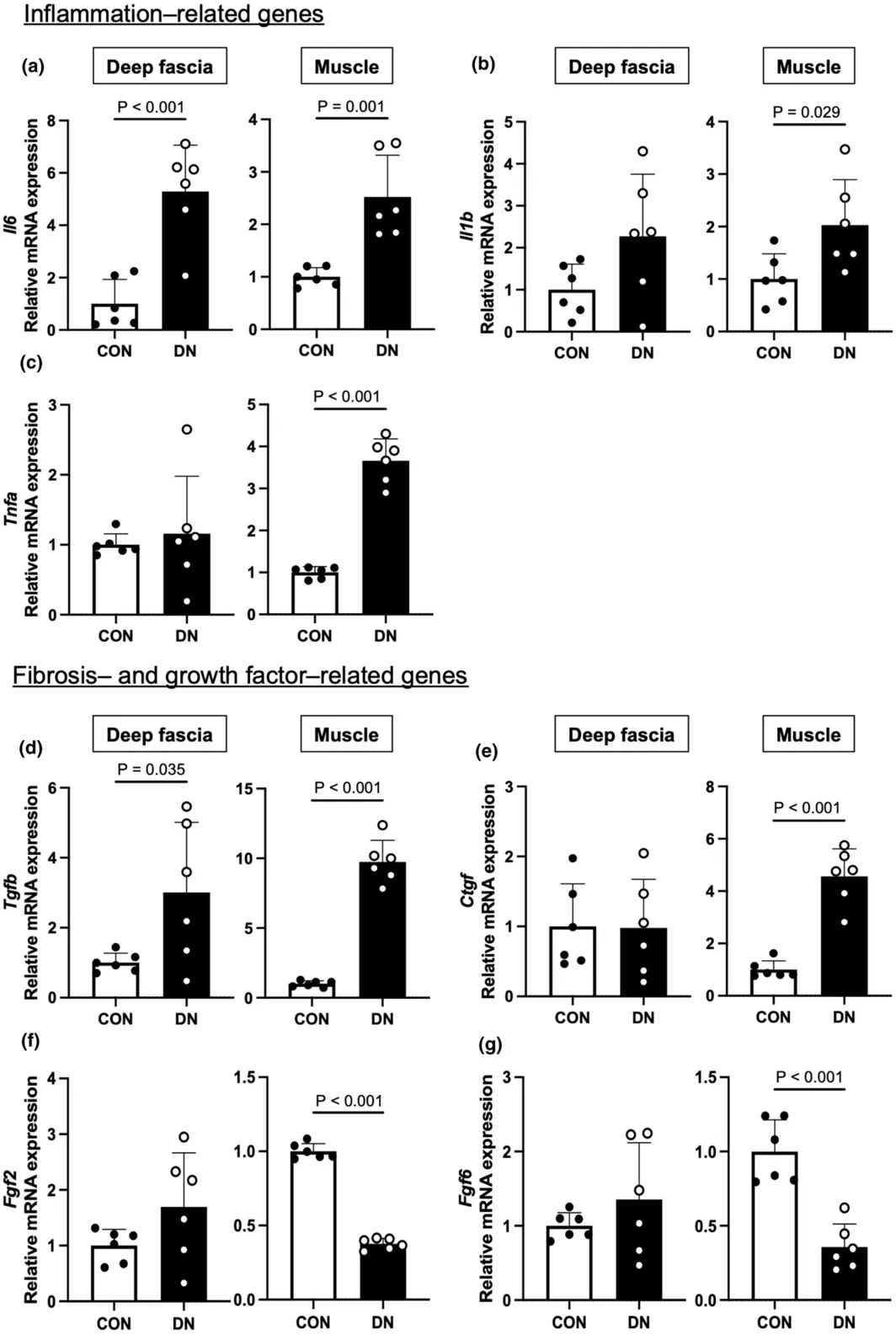
Inflammatory, fibrotic, and growth factor gene expression after denervation. Relative mRNA expression levels of inflammatory genes (a–c: Il6, Il1b, Tnfa) and fibrosis‐ and growth factor–related genes (d–g: Tgfb, Ctgf, Fgf2, Fgf6) in the deep fascia and tibialis anterior (TA) muscle of control (CON) and denervated (DN) mice. Data are presented as mean ± SD (*n* = 6/group).

Inflammation–, fibrosis–, and growth factor–related genes were differentially regulated in the DN group (Figure 3). *Il6* expression level was significantly higher in both the deep fascia (*p* < 0.001) and muscle (*p* = 0.001) (Figure 3a). In the muscle, *Il1b* and *Tnfa* expression levels were significantly higher in the DN group (*p* = 0.029 and *p* < 0.001, respectively), whereas no significant difference was observed in deep fascia (Figure 3b,c). *Tgfb* expression level was significantly higher in both the deep fascia (*p* = 0.035) and muscle (*p* < 0.001) (Figure 3d). *Ctgf* expression level was unchanged in the deep fascia but was significantly increased in muscle (*p* < 0.001) (Figure 3e). *Fgf2* and *Fgf6* expression levels did not differ between groups in the deep fascia, whereas they were significantly lower in muscle in the DN group (*p* < 0.001) (Figure 3f,g).

**FIGURE 4:**
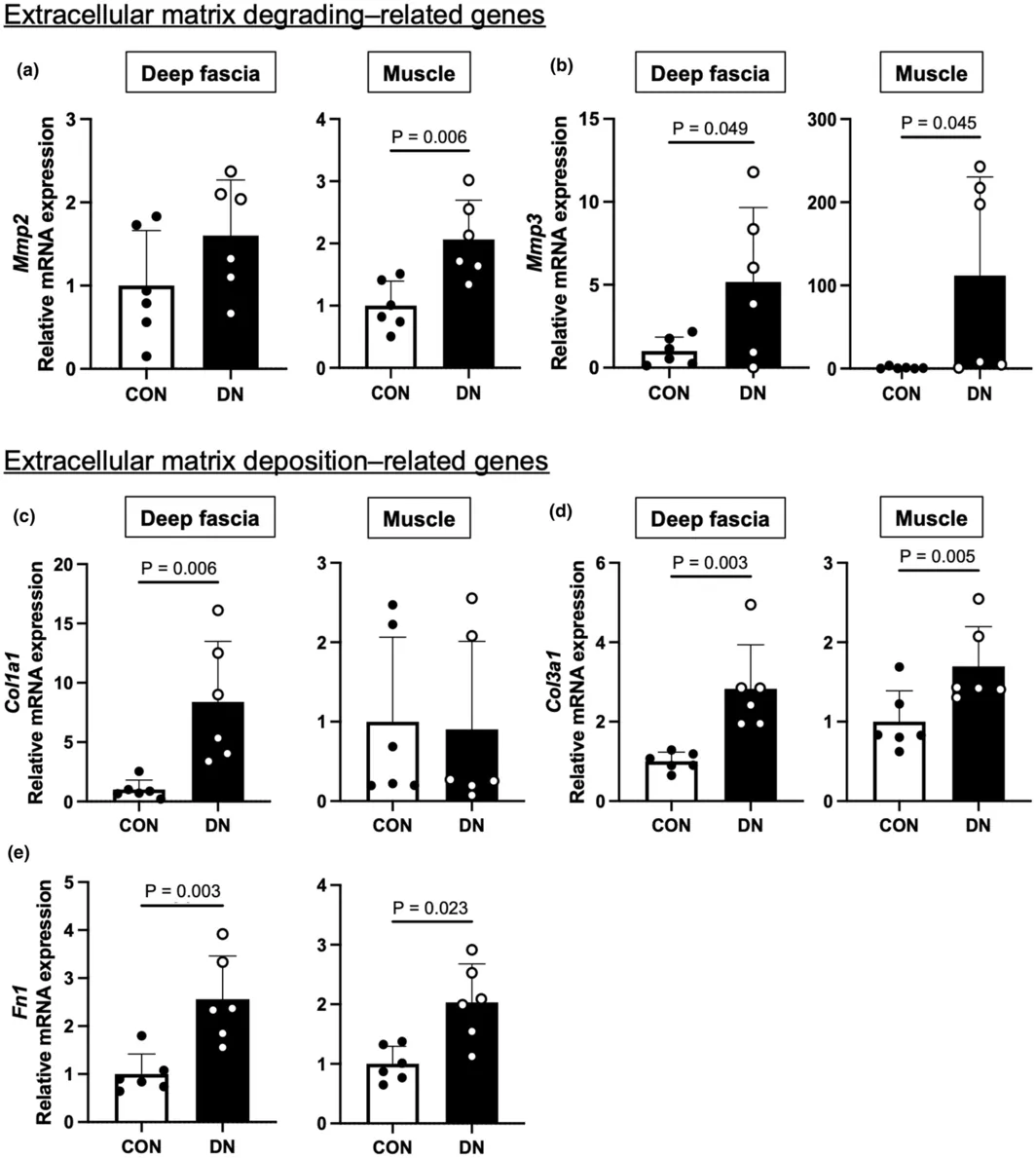
Extracellular matrix–related gene expression after denervation. Relative mRNA expression levels of extracellular matrix (ECM) degradation‐related genes (a, b: Mmp2, Mmp3) and ECM deposition‐related genes (c–e: Col1a1, Col3a1, Fn1) in the deep fascia and tibialis anterior (TA) muscle of control (CON) and denervated (DN) mice. Data are presented as mean ± SD (*n* = 6/group).

Genes related to extracellular matrix degrading and deposition also showed tissue‐specific responses (Figure 4). *Mmp2* expression level did not differ between groups in the deep fascia, whereas it was significantly higher in muscle in the DN group (*p* = 0.006) (Figure 4a). *Mmp3* expression level was significantly higher in both the deep fascia (*p* = 0.049) and muscle (*p* = 0.045) in the DN group (Figure 4b). *Col1a1* expression level was significantly higher in the deep fascia in the DN group (*p* = 0.006), whereas no significant difference was observed in muscle (Figure 4c). Expression levels of *Col3a1* and *Fn1* were significantly higher in both the deep fascia (Col3a1, *p* = 0.003; Fn1, *p* = 0.003) and muscle (Col3a1, *p* = 0.005; Fn1, *p* = 0.023) in the DN group (Figure 4d,e).

### 3.3 Collagen type I and III area fraction in the deep fascia after 14 days of sciatic denervation

**FIGURE 5:**
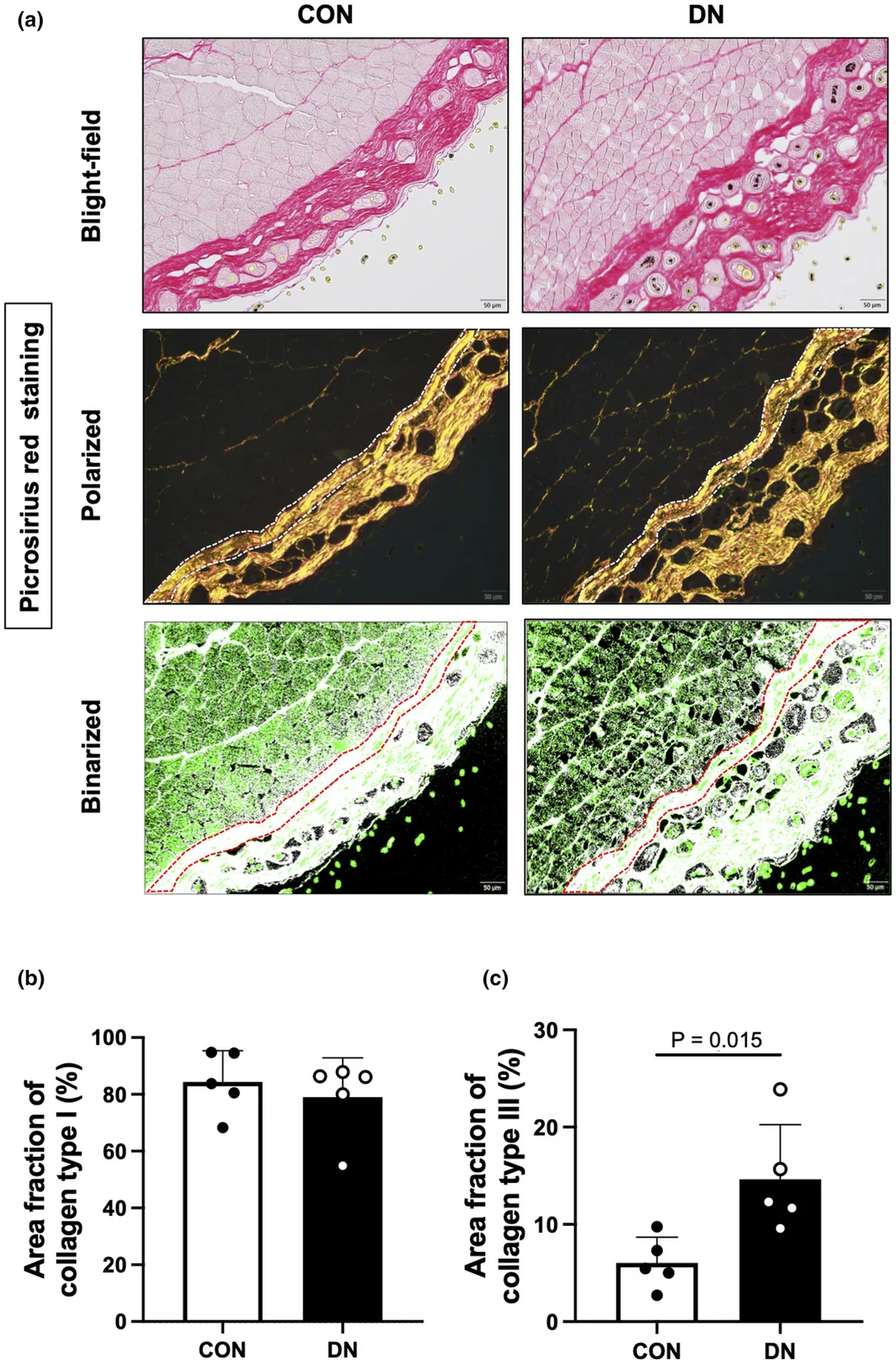
Collagen composition in the deep fascia following denervation. (a) Representative Picrosirius red staining images under bright‐field and polarized light, with corresponding binarized images used for quantitative analysis of collagen composition in the deep fascia. Scale bar = 50 μm. (b, c) Quantification of the area fraction of collagen types I and III. Data are presented as mean ± SD (*n* = 5/group).

The area fraction of collagen types I and III was assessed using PSR staining (Figure 5a). The area fraction of collagen type I in the deep fascia did not differ between the DN and CON groups (Figure 5b). In contrast, the area fraction of collagen type III in the deep fascia was significantly higher in the DN group than in the CON group (*p* = 0.015) (Figure 5c).

## 4 DISCUSSION

The present study demonstrates that sciatic nerve denervation results in significant thickening of the deep fascia accompanied by extracellular matrix (ECM) remodeling. To our knowledge, this is the first study to report both morphological and molecular alterations in the deep fascia, with direct comparison of skeletal muscle and the overlying fascia.

In the present study, we used a sciatic denervation model for 14 days, a well‐established model of disuse that induces rapid skeletal muscle atrophy and gene expression changes (Madaro et al., 2018; Umezu et al., 2021). In denervated TA muscle, we observed marked atrophy along with upregulation of inflammatory cytokines (*Il6*, *Il1b*, and *Tnfa*), fibrosis (*Tgfb* and *Ctgf*), and multiple ECM degrading (*Mmp2* and *Mmp3*) and deposition (*Col1a1* and *Fn1*) related markers, whereas several growth factors for muscle regeneration (*Fgf2* and *Fgf6*) were suppressed. This transcriptional profile is consistent with inflammation‐driven fibrosis and impaired regeneration reported in denervated skeletal muscle (Hagiwara et al., 2008; Madaro et al., 2018; Rebolledo et al., 2019; Sasabe et al., 2017). Interestingly, 14 days of denervation altered not only muscle mass but also deep fascia thickness by approximately 1.5‐fold. While previous studies have attributed connective tissues and deep fascia adaptation to mechanical loading and strain‐induced regeneration (Longrak et al., 2024; Solomonow, 2012; Tenberg et al., 2022), accumulating evidence suggests that fascia is also responsive to biochemical and inflammatory signals independent of mechanical stimulation (Langevin et al., 2011; Schleip et al., 2012; Stecco et al., 2016). The present findings demonstrate that the morphological changes of deep fascia can occur even under reduced mechanical loading, indicating that mechanical loading alone may not fully explain deep fascia remodeling.

Despite the reduced mechanical loading, the deep fascia exhibited a transcriptional response partially overlapping with that of skeletal muscle. In addition to increased expression of *Il6* and *Tgfb*, the deep fascia showed upregulation of ECM deposition–related genes (*Col1a1*, *Col3a1*, and *Fn1*), including genes not elevated in skeletal muscle. Given that TGF‐β is a central regulator of ECM production and fibrosis (Farhat et al., 2012; Leask & Abraham, 2004), and IL‐6 can potentiate profibrotic signaling (Hardee et al., 2018; O'Reilly et al., 2014), these molecular changes likely promote matrix accumulation within the fascia. Furthermore, increased *Fn1* expression may provide a provisional scaffold that facilitates subsequent collagen deposition (Mayorca‐Guiliani et al., 2025; McDonald et al., 1982; Singh et al., 2010). Consistent with these transcriptional changes, histological analysis revealed an increased proportion of green birefringence, generally associated with thin collagen fibers rich in collagen type III (Junqueira et al., 1978), and qualitative alterations in the ECM composition of the deep fascia. Collectively, these findings suggest that deep fascia thickening following denervation is driven, at least in part, by ECM accumulation associated with altered remodeling dynamics.

The similarity in gene expression patterns observed between skeletal muscle and adjacent deep fascia suggests potential inter‐tissue communication. One plausible mechanism is paracrine signaling mediated by muscle‐derived cytokines and myokines. Previous studies have shown that factors such as *Il6* and *Tgfb* are suggested to activate fibroblasts via STAT3 and SMAD signaling pathways (Hardee et al., 2018; Madaro et al., 2018; O'Reilly et al., 2014). In the present study, *Tnfa* and *Ctgf* were upregulated exclusively in skeletal muscle, whereas no significant changes were detected in the deep fascia. These findings suggest that inflammatory and profibrotic signals released from denervated muscle may influence the expression of ECM deposition–related genes within the deep fascia. This interpretation is consistent with the concept that skeletal muscle functions as a secretory organ capable of modulating the extracellular environment of surrounding tissues (Pedersen et al., 2010; Rubio‐Ruiz et al., 2019). In addition, altered mechanical interactions between atrophied muscle and the surrounding deep fascia may also contribute to deep fascia remodeling. Reduced mechanical loading associated with muscle atrophy may induce adaptive changes in the deep fascia, possibly increasing its thickness and stiffness to compensate for the loss of muscular support.

**FIGURE 6:**
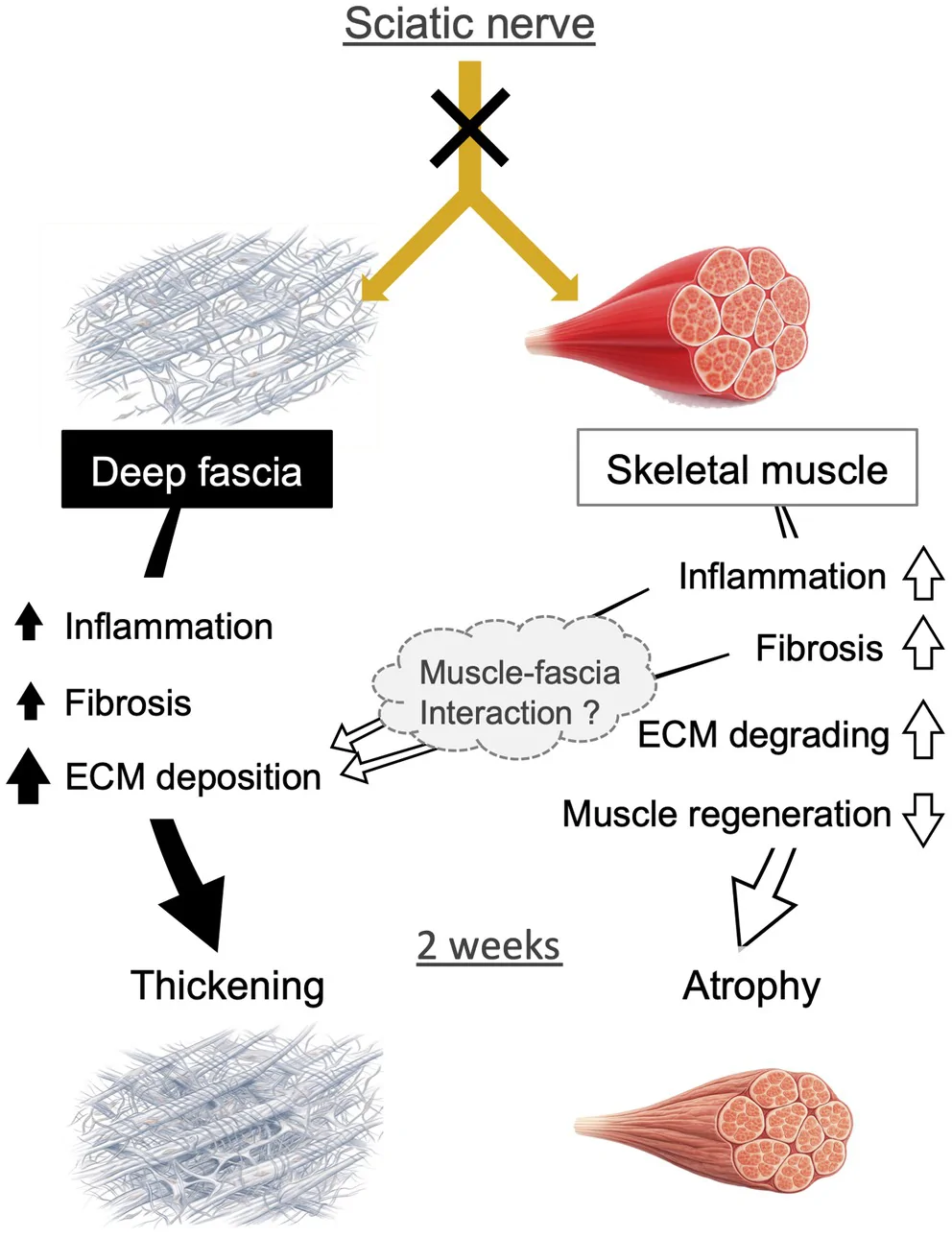
Proposed mechanism of alteration of skeletal muscle and deep fascia after denervation. Sciatic denervation induced muscle atrophy associated with increased inflammation, fibrosis, extracellular matrix (ECM) degradation, and reduced muscle regeneration. Concurrently, the adjacent deep fascia exhibited thickening together with similar but partly distinct changes in inflammation, fibrosis, and ECM deposition. This schematic illustrates the potential interactions between skeletal muscle and deep fascia during denervation‐induced tissue remodeling.

Despite partially overlapping transcriptional responses, several genes exhibited tissue‐specific regulation. For instance, *Col1a1* expression was selectively increased in the deep fascia, whereas *Fgf2* and *Fgf6* were downregulated only in the skeletal muscle. These findings indicate that, although both tissues were exposed to a denervated state, they experienced distinct biochemical and mechanical environments, resulting in tissue‐specific remodeling responses. This tissue specificity supports the concept that muscle and fascia should be considered as separate yet interacting biological compartments, and that changes in deep fascia morphology cannot be explained solely by passive spillover of muscle‐derived gene expression (Figure 6).

From a physiological and biomechanical perspective, skeletal muscle and deep fascia form an integrated system responsible for force transmission and mechanical coordination (Maas & Sandercock, 2010; Yucesoy et al., 2008). Therefore, structural alterations in the deep fascia may have important functional consequences. Specifically, increased deep fascia thickness and altered ECM composition may modify intermuscular force transmission, tissue stiffness, and mechanical coupling within the musculoskeletal system. Such changes could impair joint mechanics and movement efficiency, particularly under conditions of prolonged muscle inactivity. Clinically, these findings are relevant to conditions characterized by reduced muscle activity, including nerve injury, immobilization, and extended bed rest. Increased fascia thickness has been associated with reduced joint range of motion (Wilke et al., 2019; Wilke & Tenberg, 2020), suggesting that altered deep fascia structure may contribute to persistent functional impairment. Accordingly, rehabilitation strategies should not focus solely on muscle atrophy and strength recovery, but also consider structural and mechanical changes in the fascial tissues that may influence functional outcomes.

Several limitations should be acknowledged. First, gene expression analyses were conducted at a single time point and were limited to selected molecular markers. Second, although structural and molecular changes were demonstrated, direct biomechanical evaluation of fascia properties was not performed. Third, because sciatic nerve transection simultaneously denervates skeletal muscle and the deep fascia, the present model cannot distinguish direct effects of fascial denervation from indirect effects mediated by muscle‐derived signaling or altered mechanical loading. Future studies incorporating longitudinal analyses, comprehensive omics approaches, and mechanical testing will be essential to clarify how alterations in deep fascia structure influence force transmission and functional performance.

In conclusion, sciatic nerve denervation was associated with deep fascia thickening accompanied by extracellular matrix remodeling. These findings suggest a close but asymmetric interaction between skeletal muscle and fascia, with deep fascia alterations potentially contributing to musculoskeletal adaptation to denervation.

## AUTHOR CONTRIBUTIONS

**Shun Otsuka:** Conceptualization; data curation; formal analysis; funding acquisition; investigation; methodology; project administration; software; supervision; validation. **Yuki Tamaki:** Data curation; investigation; methodology; validation. **Kanae Umemoto:** Data curation; investigation; methodology; validation. **Naoyuki Hatayama:** Conceptualization; data curation; investigation; project administration; supervision. **Munekazu Naito:** Conceptualization; investigation; project administration; supervision.

## FUNDING INFORMATION

JSPS KAKENHI (Grant‐in‐Aid for Early‐Career Scientists), 22K17722 and 25K21005.

## CONFLICT OF INTEREST STATEMENT

The authors declare no conflicts of interest related to this study. The authors have no professional relationships with companies or manufacturers that could benefit from the results of the present study. The results of the study are presented clearly, honestly, and without fabrication, falsification, or inappropriate data manipulation. The results of the present study do not constitute endorsement by the American College of Sports Medicine.

## ETHICS STATEMENT

Experimental procedures were approved by the Institutional Animal Care and Use Committee of Aichi Medical University (Nagakute, Japan; approval number: 2023–20), in accordance with institutional guidelines and national regulations for the care and use of laboratory animals.

## Supporting information


**Table S1.** Primer sets used for gene expression analysis by RT‐qPCR.

## Data Availability

Source data for this study are not publicly available due to privacy or ethical restrictions. The source data are available to verified researchers upon request by contacting the corresponding author.
