# Supplementary material for: Sciatic denervation differentially remodels skeletal muscle and deep fascia in the lower limb of male mice
Source: Physiol Rep. 2026 Aug 20;14(16):e71063. doi: 10.14814/phy2.71063 (PMC13494361; doi:10.14814/phy2.71063)
Supplement: Supplementary file 1 [file PHY2-14-e71063-s001.docx]

**Supplementary Materials**

**Title:** Sciatic denervation differentialy remodels skeletal muscle and deep fascia in the lower limb of male mice

**Authors:** Shun Otsuka, Yuki Tamaki, Kanae Umemoto, Naoyuki Hatayama, Munekazu Naito

**Supplementary Table S1. Primer sets used for gene expression analysis by RT-qPCR.**

**Supplementary Table S1. Primer sets used for gene expression analysis by RT-qPCR.**

| Gene |  | Forward primer sequences (5'-3') | Reverse primer sequences (5'-3') |
| --- | --- | --- | --- |
| *Gapdh* |  | TGTGTCCGTCGTGGATCTGA | TTGCTGTTGAAGTCGCAGGAG |
| *Il6* |  | GCTACCAAACTGGATATAATCAGGA | CCAGGTAGCTATGGTACTCCAGAA |
| *Il1b* |  | AGTTGACGGACCCCAAAAG | AGCTGGATGCTCTCATCAGG |
| *Tnfa* |  | GGAAAGTATGTCCATTCTAAGAACAA | AGTCACTCACCAAGTAGGTTCCTT |
| *Tgfb* |  | TGGAGCAACATGTGGAACTC | GTCAGCAGCCGGTTACCA |
| *Ctgf* |  | TGACCTGGAGGAAAACATTAAGA | AGCCCTGTATGTCTTCACACTG |
| *Fgf2* |  | GGCTGCTGGCTTCTAAGTGT | GTCCCGTTTTGGATCCGAGT |
| *Fgf6* |  | GTAGAACGGGGTGTGGTGAG | CTGGGCGTTGTGTACAGTCT |
| *Mmp2* |  | ACAAGTGGTCCGCGTAAAGT | AGTCTGTGGTGGAGGAGATCA |
| Mmp3 |  | CATCCCCTGATGTCCTCGTG | CTTCTTCACGGTTGCAGGGA |
| *Col1a1* |  | TTCTCCTGGCAAAGACGGAC | CCATCGGTCATGCTCTCTCC |
| *Col3a1* |  | ACGTAAGCACTGGTGGACAG | GGAGGGCCATAGCTGAACTG |
| *Fn1* |  | ATGAGAAGCCTGGATCCCCT | GAGAGCTTCCTGTCCTGTCT |
